# A preliminary analysis of the variation in circulating 25-hydroxycholecalciferol concentrations in peri-partum spring-calving dairy cows

**DOI:** 10.1007/s11259-022-09946-z

**Published:** 2022-07-05

**Authors:** Nicholas J. Ryan, Amy Brewer, Aspinas Chapwanya, Cliona O’Farrelly, Erin J. Williams, Alexander C.O. Evans, Marijke E. Beltman, Kieran G. Meade

**Affiliations:** 1 School of Veterinary Medicine, University College Dublin, Belfield, Dublin 4, Ireland; 2 Teagasc, Animal & Grassland Research Centre, Grange, Co. Meath, Ireland; 3 Department of Clinical Sciences, Ross University School of Veterinary Medicine, St Kitts and Nevis, West Indies, Basseterre, Saint Kitts And Nevis; 4 School of Biochemistry and Immunology, Trinity College Dublin, Dublin, Ireland; 5 The Royal (Dick) School of Veterinary Studies & Roslin Institute, University of Edinburgh, Roslin, Midlothian UK; 6 School of Agriculture & Food Science, University College Dublin, Belfield, Dublin 4, Ireland; 7 Conway Institute of Biomolecular and Biomedical Research, University College Dublin, Belfield, Dublin 4, Ireland; 8 Institute of Food and Health, University College Dublin, Belfield, Dublin 4, Ireland

**Keywords:** Bovine, Cattle, Calcidiol, Immunity, Seasonal

## Abstract

Vitamin D has a well-established role in regulating the intestinal absorption of minerals but its association with immunity has not been extensively explored in livestock. Although an optimal circulating concentration of 30 ng/ml 25-hydroxycholecalciferol (25(OH)D) is proposed for immune function, it is unknown if this vitamin D concentration is sufficient, particularly for cows under a pasture-based, spring-calving dairy production system. The objectives of this retrospective analysis were to assess circulating vitamin D concentrations in a total of 843 bio-banked serum samples from Holstein-Friesian dairy cows enrolled from 12 spring-calving, pasture-based dairy farms in Ireland. Mean 25(OH)D concentrations were 36.3 ng/ml at calving, 30.7 ng/ml at 7 days post-partum (DPP), and 38.3 ng/ml at 21 DPP. However, mean concentrations masked significant inter-farm and inter-individual variation (*P* < 0.05). In fact, the proportion of cows with vitamin D insufficiency of < 30 ng/ml was found to be 33.8%, 55.5% and 19.5% at each time point, respectively. In addition, 25(OH)D concentrations correlated positively with immune cell populations (monocytes and lymphocytes) and negatively with blood urea and non-esterified fatty acids (NEFA) at 7 DPP. This is the first report of 25(OH)D concentrations in pasture-based peripartum dairy cows and we show a high degree of variation across farms and between individual animals. Sub-optimal concentrations of vitamin D in some post-partum cows may predispose cattle to multiple metabolic or infectious diseases, and therefore further work is now warranted.

## Introduction

Optimal immune function is critical to peak cow production and reduced susceptibility to disease. Multiple studies have shown dynamic changes in immune cell (Crookenden et al. 2016) and gene and protein expression (Chapwanya et al. 2009) that have important relevance for infectious and metabolic disease susceptibility (Brewer et al. 2020; Alhussien et al. 2021). This and related work has proposed that events occurring early in the post-partum period, around 7 days post-partum (DPP) are key to the early resolution of physiological inflammation and the restoration of reproductive function (LeBlanc 2014). This is a particular challenge within the tight time frame of a seasonal, predominantly grass-based system which aims to maximise the dietary intake from grass as the most cost-effective food source, and to maintain a compact calving system of a calf per cow per year.

Previously appreciated for its role in calcium homeostasis and bone development (Christakos et al. 2011), vitamin D is now emerging as an important regulator of the immune response. Few studies have been carried out in cattle, but vitamin D has been shown to regulate macrophage function in dairy cows (Corripio-Miyar et al. 2017) and also modulates the mammary immune response (Tellez-Perez et al. 2012). While optimal concentrations have not been empirically defined in cattle, metabolism of vitamin D is quite similar between cattle and humans (Nelson et al. 2012). As a result, a minimum 25-hydroxycholecalciferol (25(OH)D) threshold value of 30 ng/ml in serum has been referenced in dairy research based on previous studies in humans (Holick 2007). It is likely that vitamin D concentrations have important consequences for bone development, metabolism and potentially for immune regulation but circulating concentrations of vitamin D in pasture-based dairy cows are not currently available. Here we determined vitamin D concentrations in pasture-based Holstein-Friesian dairy cows both before and after parturition and assessed their potential association with immune and metabolic traits of relevance.

## Materials and methods

### Herd information and sample collection

A total of 843 serum samples from mixed-parity Holstein-Friesian cows across 12 farms throughout the province of Leinster in Ireland were used for the analysis described. All farms employed a pasture-based, spring-calving production system (calving dates February-March) after housing the previous winter. One farm was sampled across two consecutive years (denoted farm 2 and farm 8 in the results). Supplemental concentrate (6–8 Kg/head/day) was provided on farm using commercial ration containing 2,400iu vitamin D3/Kg feed. The 305-d milk, fat, and protein yields (kg) for the lactation period before (“previous lactation”, n = 536) and after sampling (“current lactation”, n = 661) were recorded on a monthly basis during lactation and uploaded to the Irish Cattle Breeding Federation database (www.icbf.com). The study included 192 first parity cows that did not have a milk performance in the previous lactation. As a retrospective analysis on bio-banked samples, individual cow intakes were not available. Blood sampling was carried out at calving (± 3 days), 7 DPP (± 2 days) and 21 DPP (± 4 days) and collected using 9 ml lithium heparin or 9 ml serum vacutainer® tubes for metabolite analysis. The heparin tubes were inverted several times after blood was drawn to prevent clotting. In the laboratory, tubes were centrifuged at 2000 x g for 15 min at 4˚C and then the plasma/serum was aspirated and stored in cryotubes at -20˚C.

### Haematology, metabolite and 25(OH)D measurement

Within 3 h of collection, whole blood samples were assessed using an automated haematology analyser (ADVIA 2120, Bayer Healthcare, Siemens, UK) to generate total leukocyte, neutrophil, lymphocyte, monocyte, eosinophil and basophil numbers. For metabolite analysis, concentrations of glucose, non-esterified fatty acids (NEFA), β-hydroxybutyrate (BHB) and urea were measured using a Beckman Coulter AU 400 Clinical Analyzer. Glucose was measured using the hexokinase method, urea and BHB were measured using the kinetic method, whilst NEFA and calcium concentrations were measured using the colourimetric method. 25(OH)D concentrations were measured using the Eagle Bioscience 25(OH)D ELISA kit (VID3-K01, Eagle BioScience, Nashua, NH) as previously described (Nelson et al. 2016b). Standards used for the vitamin D assay were prepared using bovine serum and concentrations were independently validated commercially using LC/MS/MS by Heartland Assays (Ames IA, 50,010). Results across the 7 concentration standards from 0 to 200 ng/ml showed > 99% correlation (data not shown). For analysis, concentrations of samples were determined using a 7-point standard curve spanning concentrations as above fitted with a four parameter logistic curve. The lower limit of detection was calculated as 2.17 ng/ml.

### Statistical analysis

All statistical analysis and data representation was conducted using Graphpad PRISM 9. A One Way ANOVA with Tukey correction for multiple testing was performed to assess differences in vitamin D concentrations. A Pearson’s correlation (PROC CORR) was used to assess relationships between production and immune variables with vitamin D concentrations in SAS 9.4 (SAS Institute, Cary, NC, USA). A P-value of < 0.05 was considered statistically significant. Data was graphed using Graphpad PRISM 9.

## Results and discussion

### Substantially lower 25(OH)D concentrations in Spring-calving, pasture-based Holstein-Friesian cows compared to more intensively managed dairy cows

**Fig. 1:**
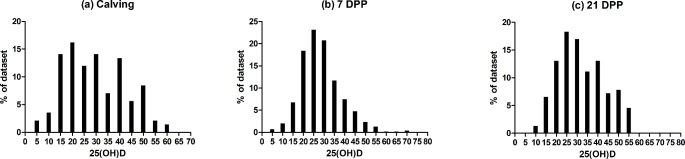
Frequency distribution of circulating 25(OH)D concentrations (ng/ml) in pasture-based, mixed-parity Holstein-Friesian dairy cows from 12 farms sampled across three time points: (a) calving, (b) 7 DPP and (c) 21 DPP. Concentrations within 5 ng of the concentration range is shown on the x-axis (E.g. the bar labelled as 5ng/ml 25(OH)D corresponds to cows with a concentration of 0–4.99 ng/ml)

The 25(OH)D metabolite in serum is the best indicator of vitamin D status as concentrations are reflective of both UVB mediated synthesis of previtamin D3 and dietary intake; and is relatively stable over time (Hymoller and Jensen 2017). The distribution of circulating concentrations is shown for the cows sampled in this study across three peripartum time points in Fig. 1**(a-c)**. Mean 25(OH)D concentrations across all farms in this study were calculated as 36.3 ng/ml at calving, 30.7 ng/ml at 7 DPP, and 38.3 ng/ml at 21 DPP (Table 1).

**Table 1:** Summary statistics for vitamin D (25(OH)D concentrations on (A) 5 farms at calving, 7 DPP and 21 DPP, total samples = 141, 169 and 153 at each time point, respectively; (B) an additional 7 farms at 7 DPP, n = 380; and (c) All farms (Total n = 843)

A Farm	1				2				3				4				5		
	Calving	7 DPP	21 DPP		Calving	7 DPP	21 DPP		Calving	7 DPP	21 DPP		Calving	7 DPP	21 DPP		Calving	7 DPP	21 DPP
n	19	24	19		72	87	84		23	26	23		15	15	15		12	17	12
Mean	34.36	35.15	40.75		20.02	20.99	24.64		38.01	35.91	39.4		47.71	45.15	42.14		41.52	43.18	44.7
SD	8.494	10.08	7.446		6.916	8.082	6.996		7.712	8.351	8.784		7.974	11.41	7.103		8.183	11.01	7.539
Minimum	18.89	16.83	28.44		5.232	3.653	9.296		26.23	21.18	17.67		28.78	30	30.1		26.6	24.13	28.86
Maximum	52.12	54	52.53		40.92	56.01	50.53		57.62	55.5	56.73		57.83	72.46	54.17		49.74	71.35	53.75
Coefficient of variation (%)	24.72	28.67	18.27		34.54	38.51	28.39		20.29	23.25	22.29		16.71	25.28	16.86		19.71	25.49	16.87
PPT < 30 ng/ml %	36.84	33.33	5.26		91.67	89.66	79.76		17.39	23.08	4.35		6.67	6.67	0.00		16.67	5.56	8.33

**Table:** 

B Farm		6		7		8		9	10	11	12
n		32		46		45		42	134	33	48
Mean		25.36		28.82		22.19		31.07	30.89	24.33	25.31
SD		6.95		6.71		3.901		9.73	8.36	5.47	5.18
Minimum		12.21		17.15		11.76		12.91	11.27	13.73	14.32
Maximum		39.36		42.46		30.36		58.19	52.03	35.29	36.07
Coefficient of variation %		27.4		23.29		17.58		31.33	27.08	22.48	20.46
PPT < 30 ng/ml %		71.88		63.04		97.78		52.38	52.99	87.88	81.25
** C** All farms											
Time	Calving		7 DPP		21 DPP						
n	141		549		153						
Mean	36.32		30.70		38.33						
SD	7.86		7.94		7.57						
Minimum	5.23		3.65		9.30						
Maximum	57.83		72.46		56.73						
PPT < 30 ng/ml %	33.85		55.46		19.54						

These values are considerably lower than what has been previously reported for dairy cows, albeit under more intensive dairy systems where supplementation is significantly higher. In a study of samples collected from cows across various stages of lactation, housing systems, and locations in the United States, average vitamin D concentrations across 12 dairy farms was reported as 68 ng/ml (Nelson et al., 2016a). Another study on almost 200 cows across 5 US dairies found highest 25(OH)D concentrations at dry off (99.7 ng/ml) (Holcombe et al. 2018).

Circulating vitamin D concentrations vary due to multiple contributory factors, including genetics and the environment (Weir et al. 2017). As sunlight is the predominant source of the precursors of active vitamin D, significant effects of both season and geographical location have been reported (Casas et al. 2015; Nelson et al. 2016b). In this study, all cows are emerging from a period of housing during the winter months, and therefore reduced exposure to sunlight is likely to be a critical contributor to the low 25(OH)D concentrations detected. This is supported by our recent findings in spring-born Holstein-Friesian dairy calves (Flores-Villalva et al. 2021).

### Significant inter-farm variation in circulating 25(OH)D concentrations in Spring-calving holstein-friesian dairy cows

**Fig. 2:**
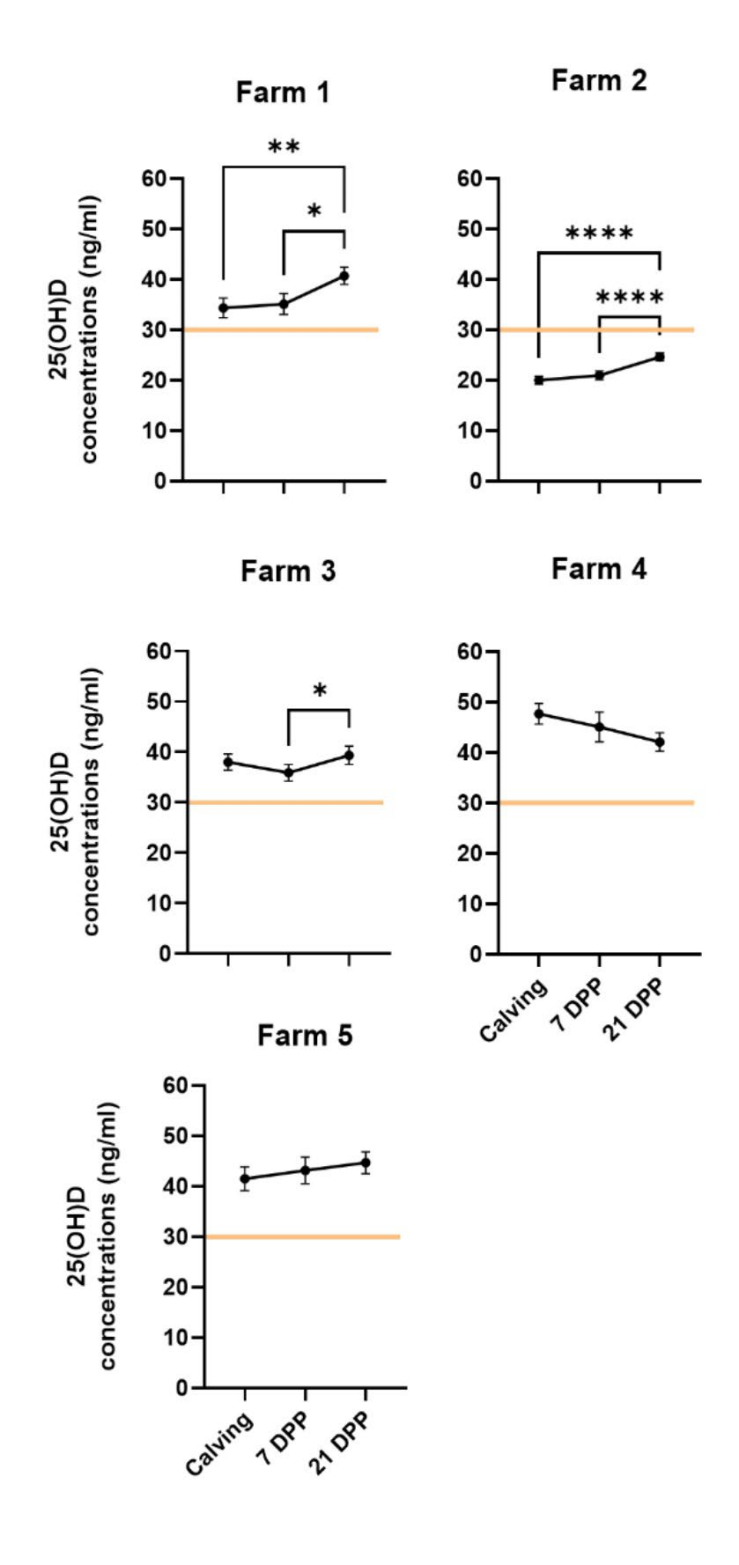
Inter-farm variation in 25(OH)D concentrations during peri-partum period: A total of 463 samples were collected across 5 spring-calving dairy farms and circulating 25(OH)D concentrations (ng/ml) were measured by ELISA across three time points: (a) calving (n = 141), (b) 7 DPP (n = 169) and (c) 21 DPP (n = 153). Data presented as mean 25(OH)D concentration (± SEM). P values are denoted as *<0.05; **<0.01; ***<0.001; ****<0.0001. The horizontal line denotes the threshold concentration currently regarded as required for 25(OH)D sufficiency

Average 25(OH)D concentrations per farm ranged from 20.0 to 47.7 ng/ml at calving, 21.0 ng/ml − 45.2 ng/ml at 7 DPP and 24.6 ng/ml – 44.7 ng/ml at 21 DPP (Table 1). An analysis of 25(OH) concentrations across the peri-partum time points showed significant changes on three out of the five farms assessed (Fig. 2). On these farms, significant increases were detected in concentrations at 21 DPP relative to earlier time points (*P* < 0.05). However individual farm data showed a high degree of variation with coefficients of variation ranging from 16.8 to 38.5% (Table 1a). These farm -specific changes likely reflect individual farm management practices including feed supplementation strategies.

**Fig. 3:**
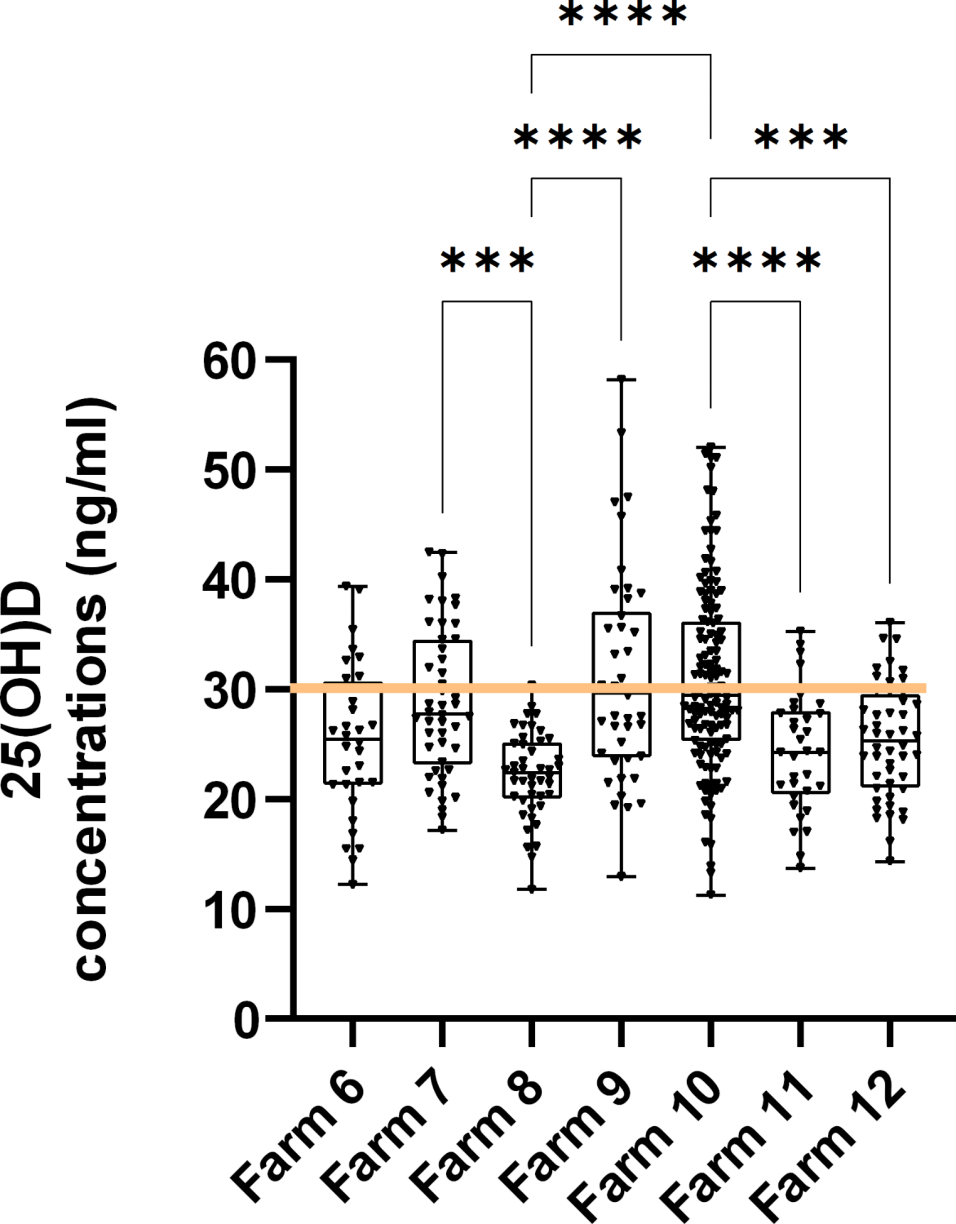
Box-plots plots showing inter-farm and inter-individual variation in 25(OH)D concentrations at 7 DPP: A total of 380 samples were collected across an additional 7 spring-calving dairy farms and circulating 25(OH)D concentrations (ng/ml) were measured by ELISA at 7 DPP. Data presented as mean 25(OH)D concentration (± SEM). For clarity only the following P value thresholds are shown ***<0.001; ****<0.0001. The horizontal line denotes the threshold concentration currently regarded as required for 25(OH)D sufficiency

Assessment of an additional 380 cows from 7 farms showed high inter-farm variation (Fig. 3). Mean concentrations were considerably lower at the 7 DPP time point and varied between 22.19 ng/ml up to 31.0 ng/ml (Table 1b).

Taking all data into consideration, the lowest overall 25(OH)D concentration of 30.7 ng/ml was observed at 7 DPP (Table 1c). Other studies have also reported a similar trend of lowest values at 7 DPP, although due to the divergence in dairy production systems, the concentrations reported are almost three-fold higher than detected here (82.6 ng/ml) (Holcombe et al. 2018).

### Significant effects of parity and milk yield on 25(OH)D concentrations

A significant effect of parity on 25(OH)D concentration was evident at each time point. The average 25(OH)D concentrations of each parity group are presented in Supplementary Table 1. The effect was strongest at calving. In comparison to primiparous cows, 25(OH)D concentrations were significantly higher in cows at lactation 3 and lactation 4. At 7 DPP, fifth lactation cows had a significantly lower circulating 25(OH)D concentration compared to than second, third and fourth lactation cows at 21 DPP (P = 0.03). At calving, multiparous cows showed significantly higher 25(OH)D concentrations than primiparous cows (43.3 ng/ml vs. 34.5 ng/ml respectively). At 7 DPP (primiparous: 31.0 ng/ml, multiparous: 30.9 ng/ml) and 21 DPP (primiparous: 38.2 ng/ml, multiparous: 38.0 ng/ml), no significant differences were apparent between groups.

The correlations between 25(OH)D concentrations and milk parameters from both lactation periods are listed in Supplementary Table 2. Calving 25(OH)D concentrations were only significantly correlated with the previous lactation’s 305-day fat kg (0.31, P = 0.04) and fat % (0.44, P < 0.01). Milk parameters from the previous lactation correlated with 7 DPP 25(OH)D concentrations included 305-day milk yield (0.11, P = 0.03), 305-day fat % (-0.12, P  = 0.03) and protein % yield (-0.17, P < 0.001). The 305d milk yield of the previous lactation correlated positively with 21 DPP 25(OH)D concentrations of 0.39 (P < 0.001). Similar correlations were also evident with 305-day milk solids (0.25, P < 0.01), fat % (0.34, P < 0.001), protein kg (-0.37, P < 0.001) and protein % (-0.33, P < 0.001).

Milk solids (0.24, P = 0.05) and fat kg (0.26, P = 0.04) were the only milk production parameters from the current lactation period that were significantly correlated with 25(OH)D concentrations at calving. Significant correlations were detected between 7 DPP 25(OH)D concentrations and 305-day milk yield (0.11, P = 0.03), fat % (-0.2, P < 0.001) and protein % (-0.12, P < 0.01). Finally, 21 DPP 25(OH)D concentrations were significantly positively correlated with 305-day milk yield (0.27, P < 0.001) and significantly negatively correlated with fat kg (-0.17, P = 0.04), fat % (-0.45, P < 0.001) and protein % (-0.38, P < 0.001).

The divergence between primiparous and multiparous groups as well as the associations with production parameters may be explained by differences in intake, particularly at calving before cows are turned out to grass and supplementation levels are highest.

### Widespread vitamin D insufficiency, particularly at 7 DPP

Vitamin D insufficiency (VDI) is defined as 25(OH)D concentrations of less than 30 ng/ml (Holick 2007; Gunville et al. 2013), and vitamin D deficiency (VDD) as concentrations less than 12 ng/ml. The average values calculated here across 12 farms here (Table 1c) obscure significant inter-individual variation. VDD was identified in five of the 12 farms sampled. At each time point, the average minimum values are indicative of VDD, with the lowest value apparent of 3.65 ng/ml at 7 DPP.

The proportion of cows below the threshold for vitamin D sufficiency (< 30 ng/ml) is shown in Fig. 3 and in Table 1. Proportions vary from a low of 19.5% of samples at 21 DPP to a high of 55.5% of farms at 7 DPP (Table 1c). Considerable inter-farm variation in VDI is also evident, with 97.8% of cows on one farm below this optimal threshold (Table 1a and b).

### 25(OH)D concentrations correlate with immune and metabolic traits at 7 DPP - a potential risk factor for inflammation and disease

Significant effects of vitamin D have been reported on diverse cell types – on mammary epithelial cells (Tellez-Perez et al. 2012; Poindexter et al. 2020) and on the activation of host defence peptides in bovine monocytes (Nelson et al. 2010; Corripio-Miyar et al. 2017) showing important immune relevance. Significant positive correlations were identified in this study between total white blood cell number (0.29, *P* = 0.013), lymphocytes (0.24, *P* = 0.039) and monocytes (0.40, *P* = 0.004) at 7 DPP (Table 2a**)**. With evidence to support a potent anti-inflammatory role in other species, vitamin D could hold significant promise for regulation of inflammation in the post-partum cow (Brewer et al. 2020).

**Table 2:** Pearson correlation analyses performed between circulating 25(OH)D concentrations (ng/ml) and (A) haematological immune cell measurements in whole blood and (B) metabolites measured in serum. ^1^Number of pairs of data points available for calculation of correlation coefficient

A.	Haematology	Total white blood cells	Neutrophils	Lymphocytes	Monocytes	Eosinophils	Basophils
	R	0.29	0.19	0.24	0.40	-0.036	0.16
	95% CI	0.063 to 0.48	-0.044 to 0.40	0.012 to 0.44	0.19 to 0.58	-0.26 to 0.19	-0.074 to 0.37
	*P*-value	0.013	0.111	0.039	0.0004	0.762	0.182
	n (pairs)^1^	78	74	74	74	74	74
B.	Metabolite	Glucose	Urea	BHB	NEFA	Calcium	Iron
	R	0.11	-0.27	-0.20	-0.26	0.33	0.14
	95% CI	-0.12 to 0.32	-0.47 to -0.052	-0.40 to 0.026	-0.46 to -0.044	0.065 to 0.55	-0.087 to 0.35
	*P*-value	0.35	0.0161	0.0833	0.0194	0.0159	0.2292
	n (pairs) ^1^	78	78	78	78	54	78

Similarly, a significant negative correlation between vitamin D concentrations and commonly measured indicators of metabolic function were detected at 7 DPP including urea (−0.27, *P* < 0.05) and NEFA (−0.26, *P* < 0.05). This could be reflective of cows in negative energy balance having lower intake (including vitamin D) and greater mobilisation of tissue reserves. The concentration of circulating calcium was significantly positively correlated with 25(OH)D (*P* = 0.016) [Table 2b]. A recent study, based in North America, identified that elevated vitamin D concentrations were associated with increased risk of ketosis and lower vitamin D concentrations also significantly associated with uterine disease (Wisnieski et al. 2020). It is therefore likely that VDI represents an important individual risk factors and warrants close attention on farm.

## Conclusions

This preliminary study reports the vitamin D concentrations for pasture-based dairy cows and identified significant correlations with farm, parity and lactation. A limitation of this study is the exclusion of other factors which are likely to partially explain some of the differences including vitamin D intakes via ration, temporal differences between cows with different calving dates and potentially unidentified sub-clinical health issues. However, given the concentrations reported in this preliminary study, results suggest that current vitamin D supplementation strategies in pasture-based spring-calving herds may not equip the transition dairy cow for optimal immune and metabolic function and therefore further detailed investigation is now warranted.

## Data Availability

The datasets generated during and/or analyzed during the current study are available from the corresponding author on reasonable request.
